# Postoperative Rheumatic Heart Disease Follow-Up: Creating a National Registry and First Results from Rwanda

**DOI:** 10.5334/aogh.2719

**Published:** 2020-09-09

**Authors:** Evariste Ntaganda, Emmanuel Rusingiza, Gilbert Rukundo, Loise Ng’ang’a, Bethany Hedt-Gauthier, Ziad El-Khatib, Gene F. Kwan, Ganza Gapira, Neil K. Worrall, JaBaris Swain, Abel Kagame, Cadet Mutumbira, Nathan Ruhamya, Ceeya Bolman, Jessica Sewase, Gilles Ndayisaba, R. Morton Bolman III, Harold Goldberg, Joseph Mucumbitsi

**Affiliations:** 1 Rwanda Biomedical Center, Kigali, RW; 2 University of Rwanda, College of Medicine and Health Sciences, Kigali, RW; 3 Partners In Health, Inshuti Mu Buzima, Kigali, RW; 4 Department of Global Health and Social Medicine, Harvard Medical School, Boston, US; 5 University School of Medicine, Boston, US; 6 Karolinska Institutet, Stockholm, SE; 7 Rwanda Military Hospital, Kigali, RW; 8 Healing Hearts Northwest, RW; 9 Team Heart, Inc, RW; 10 Rwanda Heart Foundation, RW; 11 King Faisal Hospital, Kigali, RW

## Abstract

**Background:**

In many developing countries, rheumatic heart disease (RHD) is diagnosed at an advanced stage and requires surgery for patient survival. However, access to cardiac surgery in this context is limited and often provided through partnerships, requiring centralized patient data systems for monitoring and follow-up.

**Objectives:**

This study used data from a national postoperative RHD registry to analyze clinical outcomes of Rwandan patients who received surgery between 2006 and 2017.

**Methods:**

The RHD registry was created in 2017 using data compiled from Rwanda Ministry of Health and RHD surgery partners. We extracted pre- and post-operative data on patients who were alive and in care. We excluded patients who died or were lost to follow-up, as their data was not collected in the registry. We evaluated the association between demographic, surgical, and follow-up characteristics and most recent patient symptoms, categorized by New York Heart Association (NYHA) class.

**Findings:**

Among the 191 patients eligible for inclusion in this study, 107(56.0%) were female, 110(57.6%) were adults at the time of surgery (>15 years), and 128(67.4%) had surgery in Rwanda. Most patients (n = 166, 86.9%) were on penicillin prophylaxis. Of the patients with mechanical valves, 47(29.9%) had therapeutic International Normalized Ratio values. 90% of patients were asymptomatic (NYHA I) at the time of most recent visit. NYHA class was not significantly associated with any of the considered variables. The median length of follow-up for patients was four years (IQR: 2, 5 years).

**Conclusion:**

This study shows both the feasibility and challenges of creating a RHD registry 11 years after the national initiation of RHD surgeries. Most patients captured in the registry are asymptomatic; however, collecting details on patients who had died or were lost to follow-up has proven difficult. Implementing strategies to maintain a complete and up-to-date registry will facilitate follow-up for pre- and postoperative patients.

## Background

Rheumatic heart disease (RHD) contributes to a significant proportion of cardiovascular mortality in children and young adults in low-income countries (LICs) [1,2]. Globally, 233,000 deaths annually are attributed to RHD [3]. Many LICs are unable to provide cardiac surgery as part of their national health system [4]. Patients often receive their RHD diagnosis at an advanced stage, a challenge for a disease that requires significant interventions such as open heart surgery [5]. After heart surgery, RHD patients require lifelong follow-up to optimize their clinical outcomes. This follow-up includes clinical and echocardiographic review along with regular secondary penicillin prophylaxis. Those who receive a mechanical valve require anticoagulation treatment and regular International Normalized Ratio (INR) monitoring [6].

Increasingly in Africa, RHD surgeries are becoming accessible through support from external non-governmental organizations or through referral of patients for surgery in other countries with sufficient surgical resources [7]. However, little is known about the follow-up of postoperative RHD patients in LICs in general, and in sub-Saharan Africa specifically. The few published studies report a large proportion of patients who are lost to follow-up and who receive inadequate anticoagulation treatment [1,4,8]. This highlights the need for national RHD registries to centralize RHD patient information across institutions and countries. Such systems can facilitate disease management, improve quality of care and clinical outcomes, and implement long-term monitoring to prevent loss to follow-up. In addition, these registries can be leveraged in the surveillance of acute rheumatic fever and RHD in these countries.

RHD is one of the most common heart diseases in Rwanda and, like in other African countries, Rwanda has limited capacity for surgical treatment of RHD [9,10]. This is due to the need for specific equipment, blood products, medications, and a specialized health workforce in order to provide RHD surgical services. Cardiac surgery became available in Rwanda in 2006 through partnerships between the Rwanda Ministry of Health (RMOH) and international medical humanitarian organizations [11]. In 2018 there was one full-time cardiologist working in the public sector, three cardiologists working in the private sector with some part-time support to the public sector, and three retired cardiologists offering part time support to both the private and public sectors, all of whom were based in the capital city of Kigali. Thus, RHD patients in Rwanda have remained reliant on surgical care provided through partnerships with international cardiac surgery organizations from high-income settings. In addition, some Rwandan RHD patients requiring critical valvular surgery have been referred abroad – to India, Sudan, Egypt, and other countries – with funding from the RMOH or other donors or organizations [7]. In order to increase access to care for RHD patients, the RMOH decentralized postoperative RHD care from referral facilities to district hospitals as part of the non-communicable disease (NCD) management package [12].

In 2017, the Rwanda Biomedical Centre (RBC) within the RMOH initiated a postoperative RHD registry to coordinate care and ensure availability of data to inform policy and practice in the management of RHD patients. The first phase focused on RHD patients who had previously received heart surgery, capturing postoperative details on these patients. This paper presents the early development of this postoperative RHD registry in Rwanda and includes a description of Rwandan RHD patients, both adults and children, who received surgery between 2006 and 2017 either through specialist visiting teams or through out-of-country referrals. While some of the details of surgery and follow-up of specific subgroups have been described elsewhere [7,11], this research is novel in that it describes all patients included in the newly established postoperative RHD. Additionally, this paper elucidates the process, benefits, and challenges of creating a national postoperative RHD registry 11 years after RHD surgical treatment became available in Rwanda.

## Methods

### RHD surgery and follow-up in Rwanda

In Rwanda, RHD heart surgery candidates are referred from district hospitals to cardiologists at the public teaching hospitals for preoperative screening. The pre-operative screening process is intended to determine the patient’s eligibility for surgery as well as the urgency of the need for surgery. This is based on the pre-operative symptom class, age, and likelihood of patient’s return to an asymptomatic state. Due to the limited number of heart surgeries that can be performed, the screening criteria is designed to identify the patients for whom surgery will have the maximum impact. Eligible candidates are then transferred to King Faisal Hospital (KFH), a tertiary referral hospital in Kigali. Patients who receive surgery undergo most of their treatment at KFH, which is funded through international humanitarian medical organizations in collaboration with the RMOH [7]. Following surgery, the patient remains admitted at KFH for one to two weeks for early postoperative care and monitoring. Patients are then discharged and receive monthly follow-up care for three months at either the teaching hospital or NCD clinic closest to their place of residency. After three months, long-term follow-up is done at the NCD clinic in the patient’s district or at the referral hospital. Patients who undergo surgery outside of Rwanda receive the same postoperative treatment.

Each district hospital has established a nurse-led NCD clinic running under the supervision of a medical officer, both of whom are trained in NCD management [12]. In addition to providing follow-up for postoperative cardiac patients, the package of integrated NCD clinic activities includes screening and management of chronic NCDs such as heart failure, diabetes, chronic respiratory diseases, and hypertension. Before September 2016, echocardiography and electrocardiography services were provided mostly at referral hospitals; however, since October 2017, these services are now available at most of the district hospital NCD clinics. District hospitals also have point-of-care INR machines to assess the anticoagulation levels of RHD patients who underwent mechanical valve replacement. In addition to management by the NCD nurse, all patients are reviewed at least every six months by a Rwandan cardiologist to assess their clinical and echocardiographic status. This is done through either a consultation in Kigali or mentorship outreach in which a cardiologist visits a district hospital NCD clinic on a monthly basis and sees patients in the clinic.

### Establishing a postoperative RHD registry

The postoperative RHD registry was established in 2017 by the Cardiovascular Diseases (CVD) Unit under the Division of NCDs at the RBC. The registry was established to help the RMOH monitor all Rwandan RHD patients and to evaluate the clinical status of postoperative patients. Stakeholders including RBC and local and visiting cardiologists determined the key clinical and demographic data elements that should be included in the registry to facilitate effective monitoring of postoperative patients. To complete the registry, RBC staff contacted all Rwanda-based cardiologists and three visiting surgical teams (Open Heart International, Team Heart Inc, and Healing Hearts Northwest) to collect diagnostic, postoperative, and follow-up data for all patients who have undergone RHD surgery since 2006. In addition, RBC staff reviewed medical charts from referral hospitals and NCD clinics at district hospitals to extract follow-up data for the registry. When possible, RBC staff also contacted patients to resolve any data gaps. Populating the registry has been a continuous activity and relevant patient information is updated annually. Collecting the most recent clinical information on patients who died or who were lost to follow-up has proven difficult in the retrospective creation of this registry. For that reason, only RHD cardiac surgery patients who were still alive as of 2017 and who have been determined to be actively receiving care were included in the registry. Notably, this is the first formal disease-specific registry established under the RMOH. However, an electronic medical record system is currently used for specific diseases, such as HIV. Other disease-specific registries are under development.

### Study design, population and variables

This is a retrospective descriptive study using data from the newly established postoperative RHD registry in Rwanda. We included all living patients, regardless of age, who underwent RHD surgery between 2006 and 2017 and were followed at teaching or referral hospitals or at district hospital NCD clinics. We excluded patients who had no data available in the registry, patients who were in the registry but had died, and patients who were in the registry but were lost to follow-up (i.e., missing clinic appointment for three consecutive months after mechanical valve insertion or for six consecutive months after biologic valve insertion). Demographic characteristics and pre- and post-operative clinical data were collected and input into the registry.

### Data analysis

We described patient demographic, surgical, and follow-up characteristics using frequencies and percentages for categorical data and medians and interquartile ranges (IQR) for continuous data. We categorized patients by age as either children (ages 0–15 years) or adults (ages > 15 years) at time of surgery. We used “Ubudehe” categories as a proxy for socioeconomic status. Ubudehe categories are used by the Rwandan government to identify individuals most in need for social services, with Ubudehe I indicating individuals who do not own a house are most in need, and Ubudehe IV indicating individuals who own large-scale businesses, work with international organizations and industries, or work as public servants and are least in need [13]. INR categories were classified as abnormally low (0–1.4), mildly low (1.5–2.4), therapeutic (2.5–3.5), mildly high (3.6–4.5), and very high (4.5+). We used Fisher’s exact test to assess associations between the patient’s demographic, surgical, and follow-up characteristics with their most recent postoperative New York Heart Association (NYHA) classification of symptoms. We used Stata v15 for all analyses.

### Ethics

The data for this project were collected as part of routine RBC programmatic activities. We received approval from the RBC to publish data based on the national registry (3345/RBC/DG/18). Given that this was a secondary analysis of de-identified data collected as part of a national program, this study did not require ethical approval.

## Results

### Description of patients and surgery

**Figure 1:**
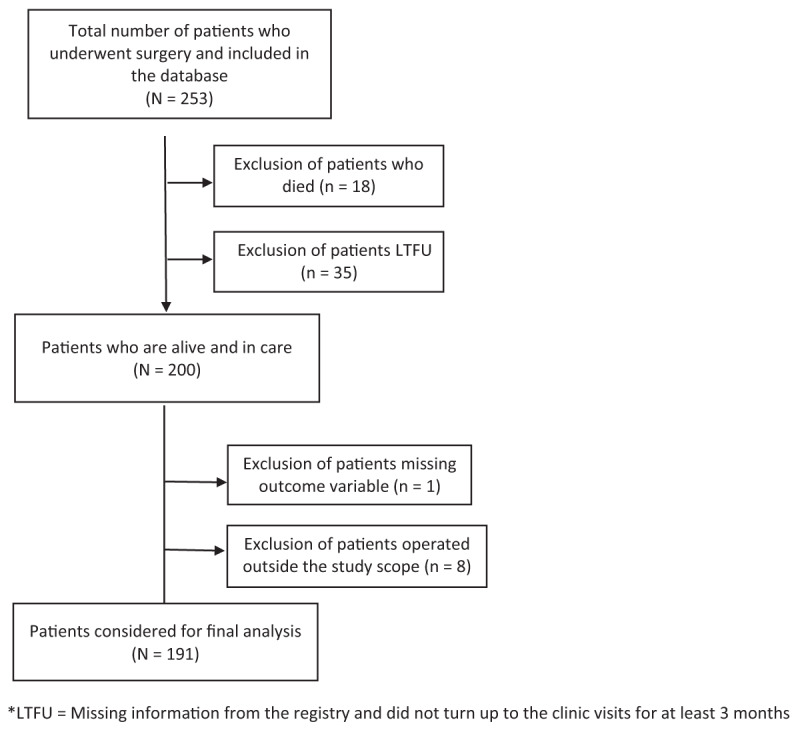
Number of patients who underwent surgery between 2006–2017: Inclusion and exclusion criteria.

Of the 253 patients in the postoperative RHD registry, 191 were eligible to be included in this study (Figure 1). Of these patients, 107(56%) were females and 110(57.6%) were adults at the time of surgery (Table 1). The median age at the time of surgery was 18 years (IQR: 13, 28 years). Patients who received surgery came from all five Rwandan provinces – the largest number resided in the Eastern Province (n = 56, 29.3%) and the smallest number resided in the Western Province (n = 24, 12.6%). Slightly more than half (n = 100, 52.3%) came from the poorest and second poorest households (Ubudehe categories I and II), 73(38.2%) were in school (either at primary, secondary, or tertiary level) at the time of the most recent follow-up visit, and 37 (19.4%) were unemployed. The majority of patients (n = 128, 67.4%) underwent surgery in Rwanda and were in either NYHA class II or III (n = 161, 84.3%) at the time of surgery. Most (n = 151, 79.9%) had mechanical valve replacement and nearly two-thirds (n = 127, 68.3%) had a single valve operation.

**Table 1:** Demographics and surgical characteristics for patients in the Rwandan Rheumatic Heart Disease National Surgery Registry.

Variable	Total	

	n	%

Province		
Eastern	56	29.3
Kigali City	36	18.8
Northern	41	21.5
Southern	34	17.8
Western	24	12.6
Gender		
Female	107	56
Male	84	44
Age category at surgery		
Adult (>15 years)	110	57.6
Children (0–15years)	81	42.4
Ubudehe Category		
Category 1 (poorest)	27	14.1
Category 2	73	38.2
Category 3	88	46.1
Category 4 (wealthiest)	3	1.6
Current Occupation		
Employed	57	29.8
In school^+^	73	38.2
Other	11	5.8
School age but not at school	13	6.8
Unemployed	37	19.4
Pre-operative NYHA		
Class 1	0	0
Class II	72	37.7
Class III	89	46.6
Class IV	30	15.7
Place of surgery*		
Rwanda	128	67.4
India	43	22.6
Other	19	10
Type of surgery**		
Bioprosthetic valve	30	16.4
Mechanical valve	151	79.2
Valve repair	8	4.4
Number of valves operated***		
One Valve	127	68.3
Two Valves	53	28.5
Three Valves	6	3.2

* 1 patient was missing data, ** 2 patients missing data, *** 5 patients missing data, ^+^ refers to patients who reported attending primary, secondary of tertiary level education on full time basis.

### Follow-up of patients

More than half (n = 100, 52.9%) of patients received their postoperative follow-up at a referral hospital (Table 2). For the 163 patients who had an indication for anticoagulation, 161 (98.8%) received anticoagulation at their most recent visit. For the 157 patients who either had mechanical or valve repair surgery and had an INR recorded at their most recent visit, 47 (29.9%) had a therapeutic INR value while 15.3% (n = 24) and 5.1% (n = 8) had abnormally low and abnormally high measures, respectively. Most patients were on penicillin prophylaxis (n = 166, 86.9%). The median length of post-surgery follow-up was four years (IQR: 2, 5 years).

**Table 2:** Post surgery follow-up details for patients in the Rwandan Rheumatic Heart Disease National Surgery Registry.

Variable	Total	

	n	%

Access to Anticoagulation (n = 163 restricted to those indicated for anticoagulation)		
Yes	161	98.8
No	2	1.2
Most recent INR value* (Restricted to mechanical/valve repair n =157)		
Abnormally low (0–1.4)	24	15.29
Mildly low (1.5–2.4)	67	42.68
Therapeutic (2.5–3.5)	47	29.94
Mildly high (3.6–4.5)	11	7.01
High (>4.5)	8	5.10
On penicillin oral and injection prophylaxis		
Yes	166	86.9
No	9	4.7
Missing	16	8.4
Years of post-surgery follow-up		
Less than 5 Years	19	9.9
5–8 Years	55	28.8
9–11 Years	117	61.3
Facility type for follow-up**		
District Hospital	86	45.5
Referral Hospital	100	52.9
Provincial Hospital	3	1.6

* 2 patients with mechanical/valve repair missing data on most recent INR. ** 2 patients missing data on facility type.

### Patient outcomes

All patients were either NYHA functional class I (n = 172, 90.1%) or II (n = 19, 9.9%) at the time of the most recent follow-up visit (Table 3). Men were marginally more likely than women to be NYHA functional class I (p = 0.080). None of the other demographic, surgical, or follow-up characteristics were associated with NYHA class (p > 0.10 for all variables) (Table 4).

**Table 3:** Most recent NYHA class by demographic, surgery and follow-up characteristics.

Variable	Most recent NYHA				

	Class I (n = 172)		Class II (n = 19)		P value

	n	%	n	%	

Gender					
Female	93	86.9	14	13.1	0.08
Male	79	94.0	5	6	
Age category at time of surgery					
Children (0–15 years)	73	90.1	8	9.9	0.978
Adult (>15 years)	99	90	11	10	
Ubudehe Category					
Category 1 (poorest)	26	96.3	1	3.7	0.532
Category 2	63	86.3	10	13.7	
Category 3	80	90.9	8	9.1	
Category 4 (wealthiest)	3	100	0	0	
Current Occupation					
Employed	48	84.2	9	15.8	0.276
In school^+^	69	94.5	4	5.5	
Other	10	90.9	1	9.1	
School age but not at School	11	84.6	2	15.4	
Unemployed	34	91.9	3	8.1	
Preoperative NYHA					
Class I	0	0.0	0	0.0	0.620
Class II	63	87.5	9	12.5	
Class III	82	92.1	7	7.9	
Class IV	27	90.0	3	10.0	
Year of surgery					
2006–2009	108	92.3	9	7.7	0.168
2010–2013	49	89.1	6	10.9	
2014–2017	15	78.9	4	21.1	
Place of surgery*					
Rwanda	117	91.4	11	8.6	0.306
India	36	83.7	7	16.3	
Other	18	94.9	1	5.1	
Type of surgery**					
Bioprosthetic valve	24	80	6	20	0.134
Mechanical valve	138	84.9	13	15.1	
Valve repair	8	100	0	0	
Number of valves operated***					
One valve	117	92.1	10	7.9	0.255
Two valves	45	84.9	8	15.1	
Three valves	6	100	0	0	
Province					
Eastern	50	89.3	6	10.7	0.253
Kigali City	35	97.2	1	2.8	
Northern	34	82.9	7	17.1	
Southern	30	88.2	4	11.8	
Western	23	95.8	1	4.2	

* 1 patient missing data, ** 2 patients missing data, *** 5 patients missing data, ^+^ refers to patients who reported attending primary, secondary of tertiary level education on full time basis.

**Table 4:** Follow up details by outcome (most recent NYHA).

Variable	Most recent NYHA				

	Class I		Class II		P value

	Freq.	Perc.	Freq.	Perc.	

Access to Anticoagulation (n = 163 restricted to those eligible for anticoagulation)					
No	2	100.0	0	0.0	0.835
Yes	147	91.3	14	8.7	
Most recent INR value (Restricted to mechanical/valve repair n = 157)					
Abnormally low	24	100.0	0	0.0	0.469
Mildly low	61	91.0	6	9.0	
Therapeutic	42	89.4	5	10.6	
Mild high	10	90.9	1	9.1	
High	7	87.5	1	12.5	
On penicillin oral and injection prophylaxis*					
No	7	77.8	2	22.2	0.233
Yes	150	90.4	16	9.6	
Years of post-surgery follow up					
Less than 5 Years	108	92.3	9	7.7	0.168
5–8 Years	49	89.1	6	10.9	
9–12 Years	15	78.9	4	21.1	
Facility^†^					
District Hospital	79	91.9	7	8.1	0.615
Referral Hospital	88	88.0	12	12.0	
Provincial Hospital	3	100.0	0	0.0	

* 16 patients missing data, ^†^ 2 patients missing data.

## Discussion

This is the first study to use the newly established national postoperative RHD registry in Rwanda and one of very few studies to describe the clinical outcomes of RHD patients who have undergone valve surgery in sub-Saharan Africa. Patients who were captured in the registry and who were alive as of 2017 were mostly children (<15 years of age) at the time of surgery and from low socioeconomic backgrounds (Ubudehe categories I and II). This is similar to demographics documented in other studies of patients undergoing RHD surgery in sub-Saharan Africa [1,7,14].

Our study confirms that postoperative follow-up for RHD patients is feasible in Rwanda. Specifically, the decentralized model of follow-up care utilizing nurse-led NCD clinics in rural district hospitals is appropriate and feasible in this setting. Nearly all living patients captured in the registry were asymptomatic (NYHA class I) and most had access to penicillin prophylaxis and anticoagulation treatment when indicated. The proportion of patients with access to penicillin (near 87%) was notably better than in other low- and middle-income countries, although it was slightly lower compared to the 93% reported in a recent study conducted in three Rwandan districts that receive intensive NGO support [9,15].

The majority of patients received cardiac surgery in-country and underwent mechanical valve replacement. Hospitals in Rwanda currently lack capacity to perform RHD-related cardiac surgeries on their own and rely on visiting humanitarian teams to perform these surgeries in-country [11]. In rural, low-income settings like Rwanda with limited access to cardiovascular services, mechanical valve replacements tend to be the preferred valve replacement due to the advanced disease at the time of diagnosis, the longevity of mechanical valves, and the reduced need for reoperation procedures in patients who receive a mechanical valve [16,17].

In addition, only a third of the patients had INR in the therapeutic range, reflecting ongoing challenges in clinical care such as lack of access to medicine or INR testing, infrequent appointments, inadequate adjustment of anticoagulation, and patient compliance issues, all of which should be explored in future studies. RBC has made strides in supporting anticoagulation services in country through the provision of clinical protocols on anticoagulation monitoring and management among post-operative RHD patients in all rural and urban NCD clinics equipped with point-of-care INR machines, as well as through training of health care workers and regular supervision of anticoagulation service by visiting cardiologists. However, more strategies must be employed to ensure proper adherence to clinical protocols by health care providers and reliable access to medications and anticoagulation treatment services for patients.

While this study shows that follow-up of RHD patients is possible in Rwanda, it also highlights the gaps in postoperative follow-up and data availability which should be considered when interpreting these results. First, because the registry is new and was developed 11 years after RHD surgeries began in Rwanda, patients who received surgery early on might be missing from the registry because they are not currently receiving care at a teaching or referral hospital or at an NCD clinic at a district hospital. Because of this, the true number of RHD patients who have received cardiac surgery is currently unknown and likely above our reported 253. In an effort to combat this for future studies, RBC will continue to update this information as more data is populated into the registry. Secondly, a third of the 253 patients identified in the registry had either died or were lost to follow-up at the time of data collection. There was little data available on the most recent outcomes of these patients and they were excluded from this analysis as a result. The exclusion of these patients introduces bias, particularly because those patients are more likely to have died or to have had poor follow-up. Finally, some variables, including complications, reproductive status, hospitalization, and cost of care either to the patients or to the national program, are not systematically collected in the registry.

The RMOH is still making efforts to identify more patients through the NCD nurses at the facility level and community health workers at the community level, as well as through external partners who have been involved in service delivery, in order to rectify missing information and enroll new and previously uncaptured patients into the registry during routine clinical follow-up. These efforts should improve the completeness, representativeness, and accuracy of the registry. The RMOH will migrate this registry to a recently developed and more complete database on acute rheumatic fever (ARF) and RHD, developed in collaboration with local cardiologists and partners. For the new registry, all data will be recorded in a REDCap (Research Electronic Data Capture) database that is hosted at RBC. REDCap is a secure web-based software platform designed to support data capture for research [18]. Data will be collected prospectively through routine patient care in district and referral hospitals in Rwanda. By collecting data prospectively, data on deaths and patients who become lost to follow-up while they are still receiving care will be captured. This will allow for a more comprehensive analyses in the future. We believe that the development of this registry will not only identify existing barriers and improve the medical management of secondary prophylaxis and post-operative RHD in Rwanda but will also provide a model for the development of other disease-specific registries contextually applicable within other programs in Rwanda.

## Conclusion

We describe the process of establishing a postoperative RHD registry in Rwanda along with results from patients tracked in this registry. Two thirds of the patients who are included in the registry to date were alive and receiving postoperative follow-up care at the time of data collection in 2017. Data on these patients demonstrate that postoperative follow-up is possible in Rwanda. However, the fact that this registry was established retrospectively, going so far back as to include patients operated on more than 10 years prior to the creation of the registry, resulted in major data limitations. A prospective postoperative RHD registry that captures all patients is likely to improve follow-up for both pre- and postoperative patients and thus improve care. Other sub-Saharan African countries should work to establish these registries as soon as possible, and once established, develop systems to continuously update patient data.
